# Long intergenic non-coding RNA 00152 promotes tumor cell cycle progression by binding to EZH2 and repressing p15 and p21 in gastric cancer

**DOI:** 10.18632/oncotarget.6949

**Published:** 2016-01-19

**Authors:** Wen-ming Chen, Ming-de Huang, Dao-ping Sun, Rong Kong, Tong-peng Xu, Rui Xia, Er-bao Zhang, Yong-qian Shu

**Affiliations:** ^1^ Department of Oncology, Jining NO.1 People's Hospital, Jining City, Shandong Province 272011, China; ^2^ Department of Medical Oncology, Huai'an First People's Hospital, Nanjing Medical University, Jiangsu Province 223300, China; ^3^ Central Laboratory, Subei People's Hospital of Jiangsu province, Yangzhou, Jiangsu Province 225001, China; ^4^ Department of Oncology, The First Affiliated Hospital of Nanjing Medical University, Jiangsu Province Hospital, Nanjing, Jiangsu Province 210029, China; ^5^ Department of Biochemistry and Molecular Biology, Nanjing Medical University, Nanjing, Jiangsu Province 210029, China

**Keywords:** long non-coding RNA, LINC00152, gastric cancer, cell proliferation, p15/p21

## Abstract

Long noncoding RNAs (lncRNAs) play important regulatory roles in several human cancers. Integrated analysis revealed that expression of long intergenic non-coding RNA 152 (LINC00152) was significantly upregulated in gastric cancer (GC). Further analysis in a cohort of 97 GC patients revealed that LINC00152 expression was positively correlated with tumor invasion depth, lymph node metastasis, higher TNM stage, and poor survival. Gene set enrichment analysis revealed that cell proliferation and cell cycle progression were increased in patients with high LINC00152 expression. In both GC cell lines and xenograft systems, LINC00152 overexpression facilitated GC cell proliferation by accelerating the cell cycle, whereas LINC00152 knockdown had the opposite effect. Moreover, by binding to enhancer of zeste homolog 2 (EZH2), LINC00152 promotes GC tumor cell cycle progression by silencing the expression of p15 and p21. These findings suggest that LINC00152 may play contribute to the progression of GC and may be an effective therapeutic target.

## INTRODUCTION

Seventy percent of all gastric cancer (GC) cases occur in developing countries, with half of all cases occurring in Eastern Asia alone (predominantly China) [1]. Unfortunately, in the early stages of GC, there are often no specific symptoms, and curative surgery is usually no longer an option at the time of diagnosis. Furthermore, treatment options are limited due to the lack of knowledge of the molecular and genetic bases of gastric carcinogenesis [2]. A deeper understanding of the molecular mechanisms of GC will shed light on its pathogenesis, and identification of new biomarkers for diagnosis and prognosis may improve individualized treatment strategies in the future [2].

Numerous genetic and epigenetic alterations are associated with GC [3–5]. Long noncoding RNA (lncRNA) is a newly-identified class of RNAs that are more than 200 nucleotides in length. lncRNAs may belong to one or more of the following five broad categories: sense, antisense, bidirectional, intronic, or intergenic [6]. lncRNA is a major component of epigenetic regulatory networks [7]. Additionally, lncRNA expression is dysregulated in GC tissues [8]. For example, over-expression of H19 promotes various aspects of GC progression, including proliferation, migration, invasion and metastasis [9]. Other lncRNAs, such as HOTAIR, colon cancer-associated transcript 1 (CCAT1), and gastric carcinoma high expressed transcript 1 (GHET1) may also play important roles in GC [10–12]. Furthermore, our previous study suggested that lncRNA TINCR regulates GC cell proliferation and apoptosis by affecting KLF2 mRNA stability [13]. Expression of another lncRNA, long intergenic non-coding RNA 152 (LINC00152), was upregulated in GC and is present in both gastric juice and plasma, suggesting that it could be used as a reliable biomarker for GC diagnosis [14]. However, its prognostic value and effects on GC progression require further investigation.

Enhancer of Zeste Homolog 2 (EZH2), the core catalytic component of the polycomb repressive complex 2 (PRC2), also affects cancer progression by altering histone H3lysine27 (H3K27) trimethylation and silencing transcription [15]. EZH2 is frequently overexpressed in GC, and EZH2 levels are positively correlated with tumor size, depth of invasion, vessel invasion, lymph node metastasis, and clinical stage [22]. Downregulation of EZH2 suppresses cell growth, migration, and invasion, and induces cell cycle arrest in GC cells [23]. Many long intergenic non-coding RNAs (LinCRNAs) regulate gene expression through interactions with EZH2 [17, 24]. For example, HOTAIR interacts with EZH2 to silence tumor suppressor genes [16]. Other lncRNAs (e.g., EBIC, H19, PVT1, and MALAT1) also interact with EZH2 in cancer cells [17–20], and may promote malignancy in GC by altering EZH2 activity.

In this study, we investigated the relationship between LINC00152 expression and GC prognosis. We then examined the effects of LINC00152 on GC cell growth both *in vitro* and *in vivo*. Finally, we tested whether LINC00152 interacts with EZH2 to affect tumor cell cycle progression by silencing gene expression. These studies will help clarify the role of LINC00152 in GC progression and its potential as a therapeutic target.

## RESULTS

### LncRNA expression profiles in gastric cancer tissues

By analyzing data (GSE13911) from the National Center for Biotechnology Information (NCBI) Gene Expression Omnibus (GEO), we identified 30 lncRNAs that were highly expressed in GC tissues as compared to normal gastric tissues (Supplementary Figure S1). The most dramatically upregulated lncRNAs in GC tissues were LL22NC03-N64E9.1, H19, MIR663AHG, RP11–172H24.4, CTB-193M12.5, RP3–428L16.2, MIR4435–1HG, LINC00152, RP11–588K22.2, and HCG11. Most of these do not have an official Human Genome Nomenclature Committee symbol, and their functions are unknown. However, some of these lncRNAs, including H19 and LIN00152, play a role in cancer. H19 affects cell proliferation and predicts poor prognosis in GC [21]. We therefore investigated the relationship between LINC00152 and GC in this study.

### LINC00152 expression is increased in human gastric cancer tissues

**Figure 1:**
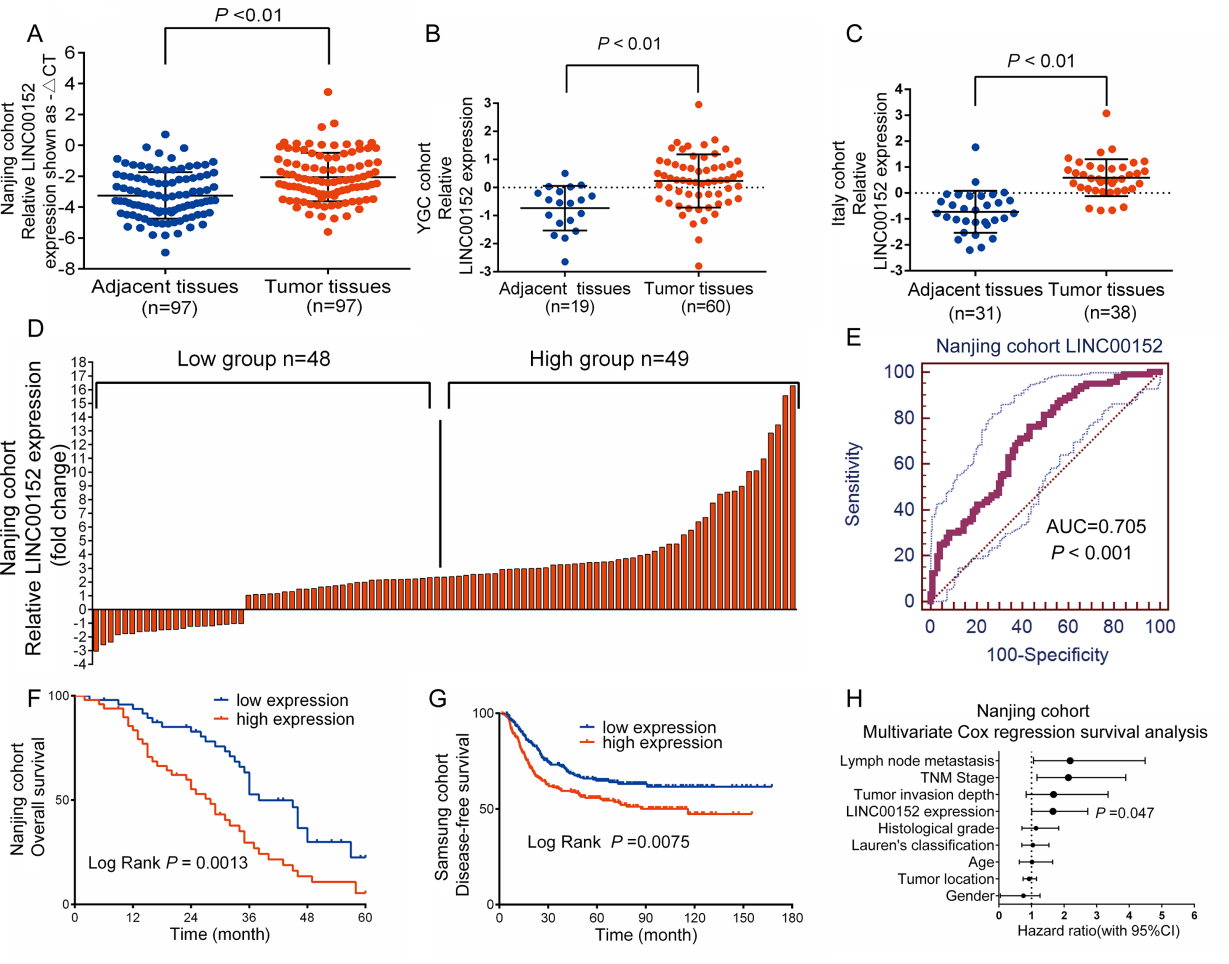
LINC00152 upregulation correlates with poor survival in patients with gastric cancer (**A**), (**B**), (**C**) Analysis of LINC00152 expression in tumor tissues compared to adjacent non-tumor tissues in 3 independent datasets (the Nanjing, YGC, and Italy cohorts). (**D**) Patients from the Nanjing cohort were assigned to one of two groups according to LINC00152 levels (median split). (**E**) The receiver operating characteristic (ROC) curves for determining the diagnostic value of LINC00152 levels. (**F**) Kaplan-Meier analysis of overall survival according to LINC00152 expression levels in the Nanjing cohort. (**G**) Kaplan-Meier analysis of disease-free survival based on LINC00152 level in the Samsung cohort. (**H**) Different factors (including LINC00152 level, tumor invasion depth, lymph node metastasis, TNM stage, histological grade, gender, tumor location, Lauren's classification, and age) were analyzed for their association with patient survival using the Cox regression model.

LINC00152 expression was upregulated in GC tumor tissues compared to adjacent non-tumor tissues in the GEO analysis (2.443-fold, *p* < 0.001, Supplementary Figure S1). To validate this result, we measured LINC00152 mRNA expression in 97 pairs of GC tissue and adjacent non-tumor tissue (Nanjing cohort) using quantitative PCR (qPCR). LINC00152 expression was higher in GC tissues than that in adjacent non-tumor tissues (*p* < 0.01, Figure 1A). The results were consistent with data sets from the GEO (Figure 1B, 1C).

### Diagnostic value of LINC00152 overexpression

A receiver operating characteristic (ROC) curve was generated by comparing LINC00152 expression in GC tissues to expression in matched adjacent non-tumor tissues. With a cutoff value of 3.175, the area under the ROC curve (ROC^AUC^) was up to 0.705 (95% CI: 0.636–0.769, *p* < 0.001, Figure 1E). The sensitivity was 76.29% and the specificity was 56.70% with a Youden index of 0.330.

### LINC00152 overexpression correlated with poor survival in GC patients

We examined the association between LINC00152 expression and various pathological clinical features in GC. 97 GC patients were assigned to one of two groups according to LINC00152 levels (median split): the high group (*n* = 49, LINC00152 expression > 2.35-fold) and the low group (*n* = 48, expression ≤ 2.35-fold) (Figure 1D). As shown in Table 1, high LINC00152 expression was positively correlated with tumor invasion depth (*p =* 0.029), lymph node metastasis (*p* = 0.047), and higher TNM stage (*p =* 0.025), but not with age, gender, histological grade, tumor location, or histological subtype.

**Table 1:** Relationships between the expression of LINC00152 and clinicalpathological characteristics in 97 patients with GC

Characteristics	Expression of LINC00152		*P* value
	Low (*n* = 48)	High (*n* = 49)	
**Gender**			0.823
Male	35	34	
Female	13	15	
**Age**			0.219
≤60	25	32	
>60	23	17	
**Histological grade**			0.418
Well/moderate	20	25	
other	28	24	
**Tumor invasion depth (T)**			0.029
Tis, T1, T2	20	10	
T3 or above	28	39	
**Lymph node metastasis (N)**			0.047
N0	14	6	
N1 or above	34	43	
**TNM stage**			0.025
I/II	32	21	
III/IV	16	28	
**Tumor location**			0.712
Antrum	17	17	
Cardia	15	11	
Angulus	12	13	
Body	2	5	
Full stomach	2	3	
**Laure's classification**			0.993
Intestinal	20	21	
Diffuse	24	24	
Mixed	4	4	

Notably, LINC00152 overexpression strongly correlated with poor survival. The median survival time in the high expression group was substantially shorter than that in the low expression group (28 vs 38 months, *p* = 0.0013, Kaplan–Meier analysis, Figure 1F). Multivariate Cox regression survival analysis adjusted for TNM stage, tumor invasion depth, sex, and patient age revealed a strong correlation between LINC00152 overexpression and shorter survival (*p =* 0.047, HR = 1.659, 95% CI: 1.008 to 2.731, Figure 1H, Table 2). This result was validated in the Samsung cohort, in which LINC00152 upregulation was also associated with poor prognosis in GC patients (*p* = 0.0075, Kaplan–Meier analysis, Figure 1G).

**Table 2:** Univariate and multivariate analysis of clinical pathological characteristics and overall survival of 97 patients with GC (Nanjing cohort)

	Univariate analysis			Multivariate analysis		
Factors	HR	95% CI	*P*-value	HR	95% CI	*P*-value
LINC00152 expression	2.162	1.327–3.524	0.002	1.659	1.008–2.731	0.047
Tumor invasion depth (T1, T2/above)	2.528	1.423–4.489	0.002	1.676	0.838–3.352	0.145
Lymphatic metastasis (absent/present)	2.611	1.290–5.286	0.008	2.187	1.065–4.489	0.033
TNM stage (I/II, III/IV)	3.261	1.985–5.357	< 0.001	2.133	1.169–3.895	0.014
Histology grade(well, moderate/others)	1.139	0.706–1.837	0.594			
Age	1.017	0.627–1.649	0.946			
Gender (male/female)	0.751	0.446–1.263	0.280			
Tumor location	0.926	0.742–1.155	0.494			
Lauren's classification	1.041	0.704–1.537	0.842			

### Cellular localization of LINC00152

**Figure 2:**
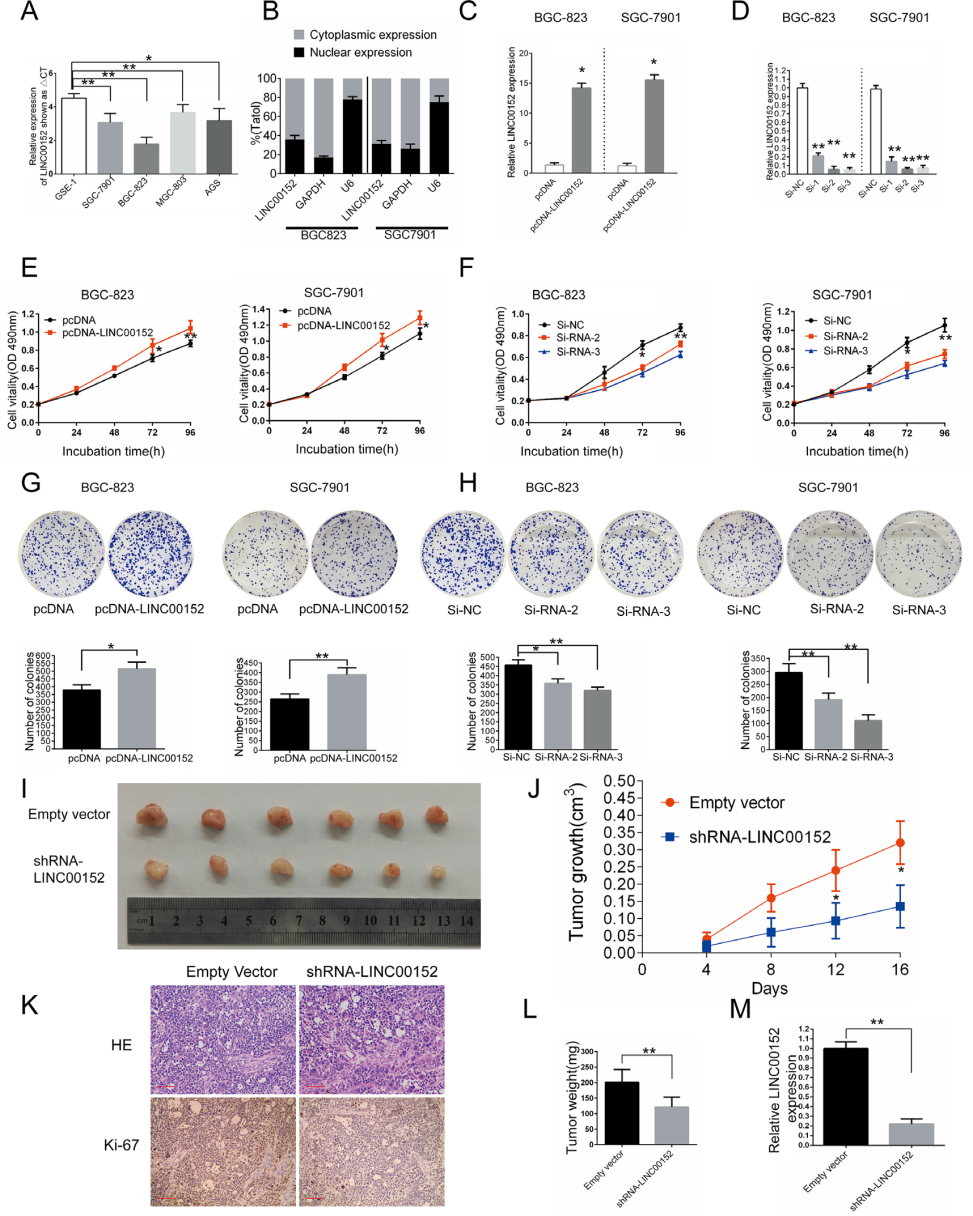
Effects of LINC00152 on gastric cell growth *in vitro* and *in vivo* (**A**) Analysis of LINC00152 expression in GC cell lines (MGC-803, BGC-823, SGC-7901, and AGS) compared to the normal gastric epithelium cell line (GES-1) by qPCR (shown as ΔCt). (**B**) Subcellular localization of LINC00152 was determined using fractionation. After nuclear and cytoplasm separation in BGC-823 and SGC-7901 cells, RNA was extracted from both fractions and LINC00152 expression was measured by qPCR. GAPDH was used as a cytoplasm marker and U6 was used as a nucleus marker. (**C**) Relative LINC00152 levels in BGC-823 and SGC-7901 cells transfected with pcDNA or pcDNA-LINC00152 were tested by qPCR. (**D**) Relative LINC00152 levels in BGC-823 and SGC-7901 cells transfected with negative control (si-NC) or LINC00152 siRNAs were tested by qPCR. (**E**), (**F**) BGC-823 and SGC-7901 cells were seeded in 96-well plates after transfection with pcDNA-LINC00152, empty vector (pcDNA), LINC00152 siRNA, or negative control, and cell proliferation was assessed daily for 4 days using an MTT assay. (**G**), (**H**) Representative colony formation assay in BGC-823 and SGC-7901 cells after transfection with pcDNA-LINC00152, empty vector (pcDNA), LINC00152 siRNA, or negative control. (**I**) Tumors from mice 16 days after injection of SGC-7901 cells stably transfected with sh-LINC00152 or empty vector. (**J**) Tumor volumes were calculated after injection every 4 days. (**K**) Tumors developed from sh-LINC00152-transfected SGC-7901 cells showed lower Ki-67 protein levels than tumors developed from control cells. (**L**) Tumor weights are represented as mean ± SD. (**M**) qPCR was performed to detect average LINC00152 expression in xenograft tumors (*n* = 6). Results are represented as the average ± SD based on 3 independent experiments (*p < 0.05, **p < 0.01).

qPCR revealed that LINC00152 mRNA expression was higher in all four human GC cell lines (BGC-823, MGC-803, SGC-7901, and AGS) than in the normal gastric cell line GES1 (Figure 2A). BGC823 and SGC7901 cells were used in subsequent studies.

To determine the cellular localization of LINC00152, we fractionated GC cells into nuclear and cytoplasmic fractions, and then separated the nucleus from the cytoplasm. Separation was confirmed by the presence of GAPDH mRNA only in the cytoplasm fraction and nuclear U6 mRNA predominantly in the nuclear fraction. qPCR showed that LINC00152 was present in both the nuclear (36.07% ± 3.98%) and the cytoplasmic fractions (63.93% ± 3.98%) of BGC-823 cells (Figure 2B). LINC00152 was also detected in both the nuclear (31.37% ± 3.33%) and cytoplasmic (68.63% ± 3.33%) fractions of SGC-7901 cells (Figure 2B).

### Relationship between cell proliferation, cell cycle regulation, and LINC00152 in GC

To analyze the biological function of LINC00152 in GC, Gene Set Enrichment Analysis (GSEA) was performed in Singapore datasets (GSE15459). GC patients were separated into two subgroups based on median LINC00152 levels. GSEA enrichment plots showed that cell proliferation and cell cycle regulation gene signatures were more strongly correlated in patients with high LINC00152 expression than in patients with low expression in GSE15459 datasets (Supplementary Figure S2A, S2B).

### LINC00152 promotes GC cell proliferation *in vitro* and *in vivo*

To validate the GSEA analysis, we transfected BGC-823 and SGC-7901 cells with LINC00152-overexpressing plasmid or LINC00152 siRNA to increase or decrease LINC00152 expression, respectively (Figure 2C, 2D). MTT assays indicated that increased LINC00152 expression promoted cell proliferation in both cell lines (Figure 2E), and LINC00152 knockdown inhibited cell proliferation (Figure 2F). A colony formation assay revealed that LINC00152 overexpression increased GC cell colony formation (BGC-823 cells: 36.0% increase, SGC-7901 cells: 47.2% increase, Figure 2G), and LINC00152 knockdown suppressed colony formation, compared to control cells (Figure 2H). An EdU assay confirmed these results and revealed that decreased LINC00152 levels suppressed cell proliferation in both BGC-823 and SGC-7901 cells (Supplementary Figure S3).

To determine whether LINC00152 affects tumor proliferation *in vivo*, we injected SGC-7901 cells transfected with either empty vector or sh-LINC00152 into male nude mice. Consistent with *in vitro* results, tumor growth in the sh-LINC00152 group was slower than in the empty vector group (Figure 2I, 2J). Sixteen days after injection, the average tumor weight in the sh-LINC00152 group (121.67±31.89 mg) was lower than that in the control group (201.33 ± 41.21 mg) (*p* < 0.01) (Figure 2L). LINC00152 expression in mouse tumor tissues was measured using qPCR (Figure 2M). Average LINC00152 expression was lower in the sh-LINC00152 than in the empty vector group. Moreover, immunohistochemistry (IHC) revealed that tumors that developed in mice injected with empty vector-transfected cells had higher Ki-67 expression than tumors in mice injected with sh-LINC00152 cells (Figure 2K). These data also suggest that LINC00152 increases GC cell growth and proliferation.

### LINC00152 regulates cell cycle in GC

**Figure 3:**
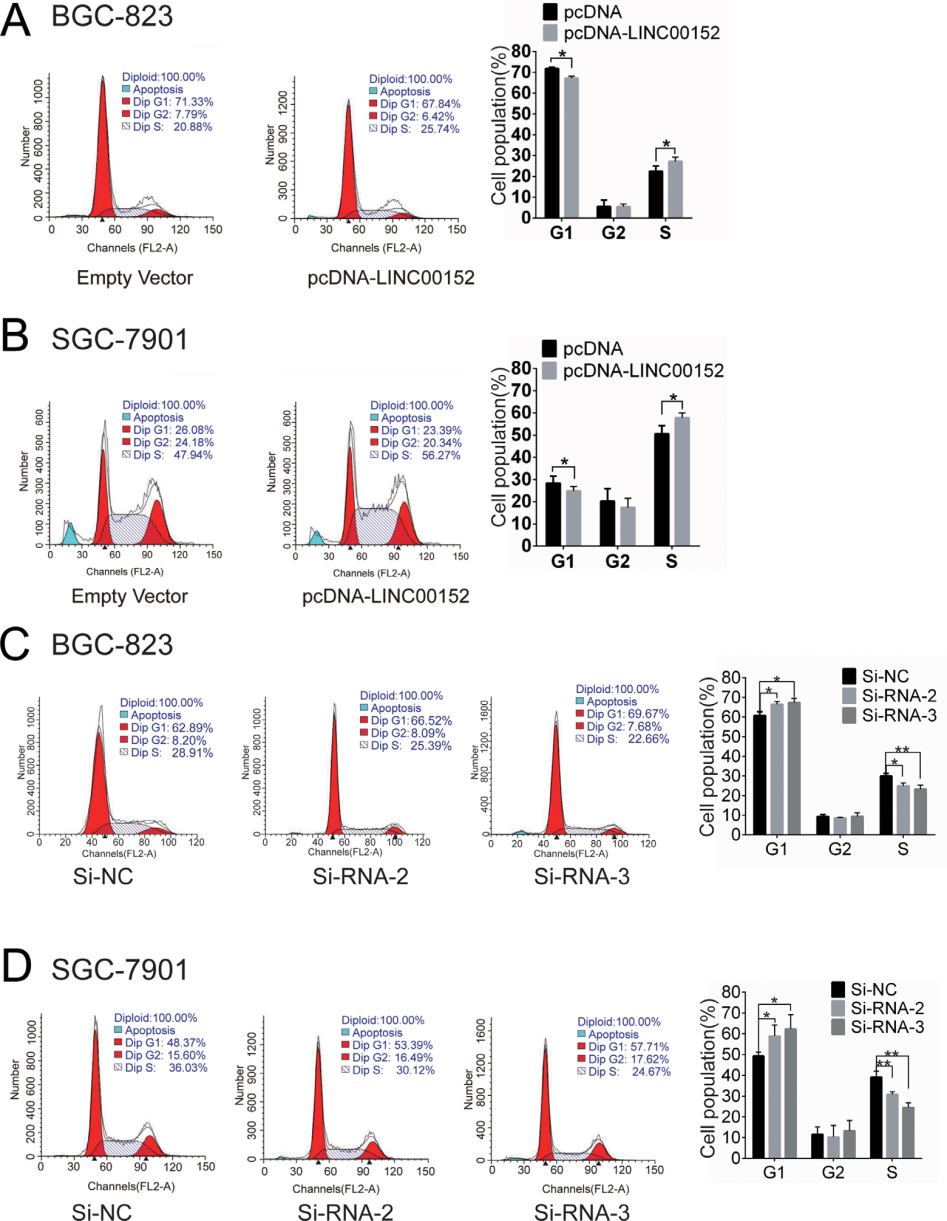
LINC00152 expression regulates GC cell cycle (**A**), (**B**) Cell-cycle analysis in BGC-823 and SGC-7901 cells after transfection with pcDNA-LINC00152 or empty vector (pcDNA). (**C**), (**D**) Cell-cycle analysis in BGC-823 and SGC-7901 cells after transfection with LINC00152 siRNA or negative control. Summarized flow cytometry data are shown. Results are represented as the average ± SD based on 3 independent experiments (*p < 0.05, **p < 0.01).

Flow cytometry showed that cell cycle arrest at the G1 phase increased in both BGC-823 and SGC-7901 cells when LINC00152 was repressed (Figure 3C, 3D). In contrast, LINC00152 overexpression promoted cell cycle progression (Figure 3A, 3B).

### LINC00152 is associated with EZH2

**Figure 4:**
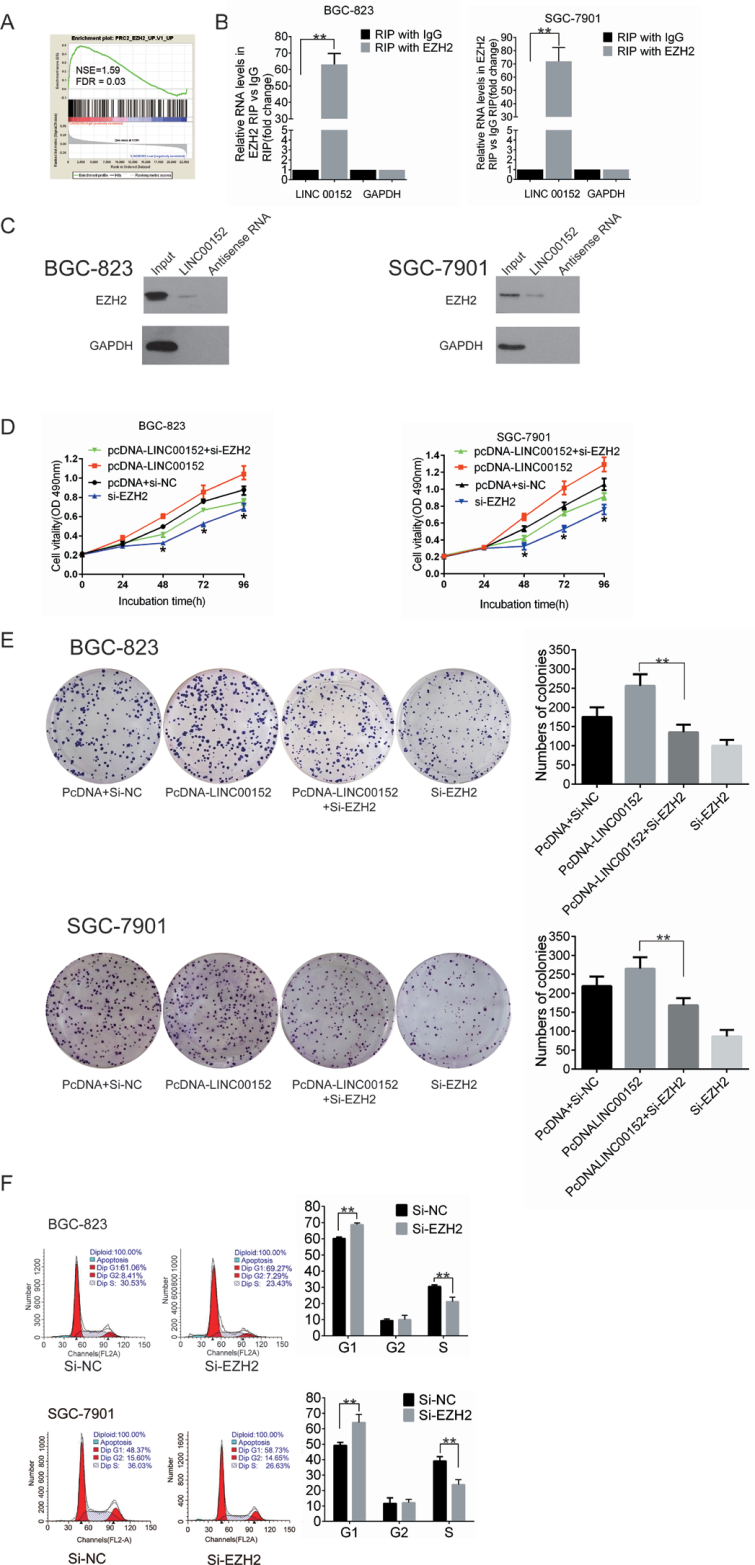
LINC00152 binds to EZH2 (**A**) GSEA comparing the high (red) and the low (blue) LINC00152 expression GC patient groups in the GSE15459 dataset, illustrating a close correlation between expression of LINC00152 and EZH2. The enrichment score (ES, green line) indicates the degree to which the gene set is overrepresented at the top or bottom of the ranked list of genes. Black bars indicate the position of genes in the ranked list of genes included in the analysis. A positive value indicates stronger correlation with high LINC00152 expression and a negative value indicates stronger correlation with low LINC00152 expression. (**B**) RIP experiments were performed in BGC-823 and SGC-7901 cells, and LINC00152 levels were analyzed in co-precipitated RNA using qPCR. The fold enrichment of LINC00152 in EZH2 RIP is relative to its matching IgG control RIP (*p < 0.05, **p < 0.01. (**C**) An RNA pull-down assay was performed as described in the experimental procedures. LINC00152 and antisense RNA was incubated with cell extracts, and EZH2 protein was assayed by Western blot analysis. A nonspecific protein (GAPDH) is shown as the control. (**D**) An MTT assay was performed to measure proliferation of BGC-823 cells and SGC-7901 cells transfected with empty vector (pcDNA) + negative control (si-NC), pcDNA-LINC00152, Si-EZH2, or pcDNA-LINC00152 + Si-EZH2. (**E**) Colony-forming growth assays were performed to measure proliferation of BGC-823 cells and SGC-7901 cells transfected with empty vector (pcDNA) + negative control (si-NC), pcDNA-LINC00152, Si-EZH2, or pcDNA-LINC00152 + Si-EZH2. (**F**) Cell-cycle analysis of BGC-823 and SGC-7901 cells after transfection with siEZH2 or negative control. Summarized flow cytometry data are shown. Results are represented as the average ± SD based on 3 independent experiments (*p < 0.05, **p < 0.01).

To determine whether LINC00152 affects gene expression by recruiting EZH2 to its target genes, we examined data downloaded from GEO (GSE15459). 200 GC patients were assigned to either the high or low expression group according to the median LINC00152 expression ratio. Gene signatures indicative of LINC00152-induced EZH2 occupancy (Normalized Enrichment Score (NES) = 1.59, False Discovery Rate (FDR) = 0.03, Figure 4A) were enriched in GC.

RNA immunoprecipitation (RIP) using an antibody against EZH2 in BGC823 and SGC7901 cells confirmed the GEO results. As shown in Figure 4B, endogenous LINC00152 was enriched in the anti-EZH2 RIP fraction compared to the IgG fraction in BGC-823 and SGC-7901 cell lines. Furthermore, an RNA pull-down assay confirmed the specificity of this interaction (Figure 4C). Together, these results demonstrate a specific association between LINC00152 and EZH2.

Additionally, endogenous LINC00152 was enriched in the anti-SUZ12 RNA immunoprecipitation (RIP) fraction in both SGC-7901 and BGC-823 cells (Supplementary Figure S4B). However, this result was not confirmed by GSEA analysis (Supplementary Figure S4A) or an RNA pull-down assay (Supplementary Figure S4C).

### LINC00152 promotes GC proliferation and cell cycle progression by binding to EZH2

To further confirm that EZH2 is involved in LINC00152-induced increases in GC cell proliferation, we performed rescue experiments (Figure 4D, 4E). Cotransfection of pcDNA-LINC00152 and si-EZH2 partially reversed the increased proliferation induced by LINC00152 upregulation, indicating that LINC00152 increases cell proliferation through EZH2. Moreover, flow cytometric analysis demonstrated that more si-EZH2-transfected cells were stalled at the G1 cell cycle phase compared to cells transfected with si-NC (Figure 4F).

**Figure 5:**
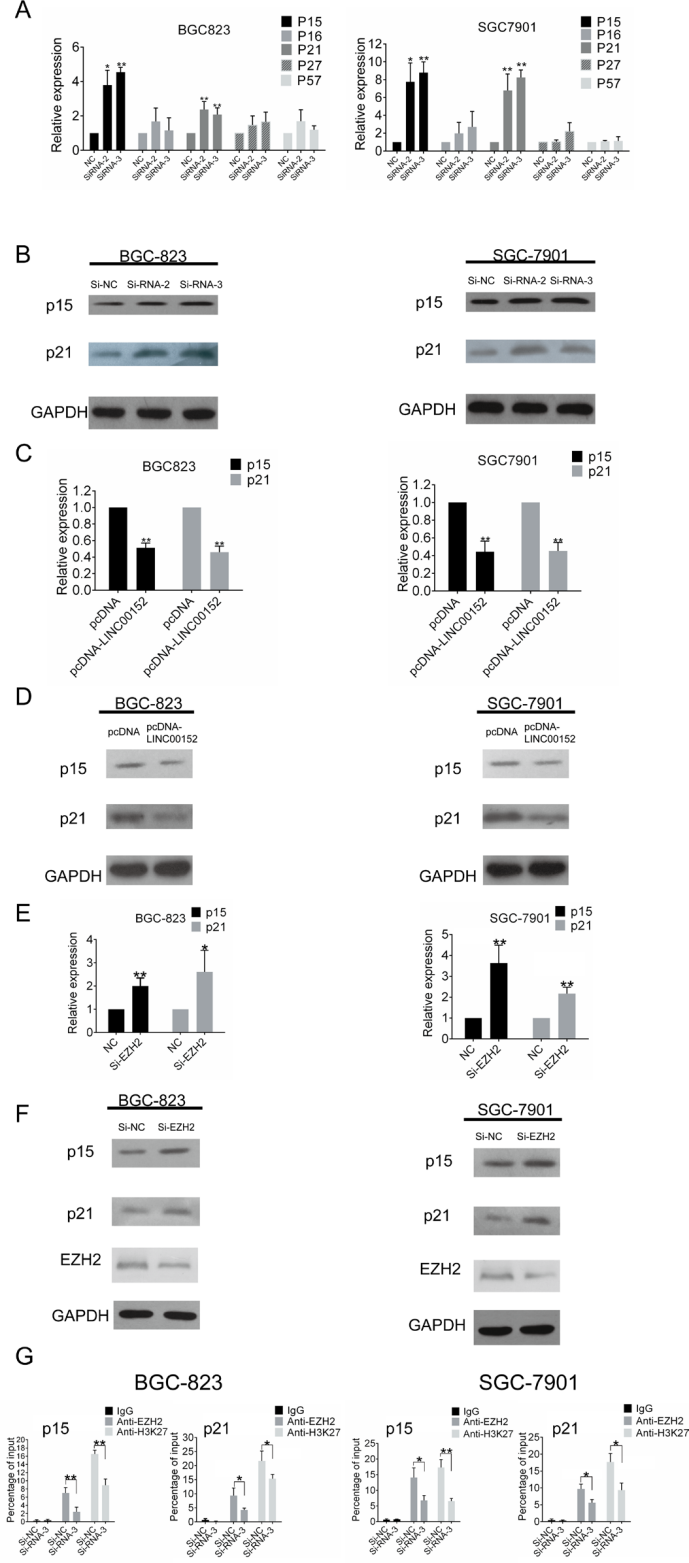
LINC00152 regulates p15 and p21 expression by binding to EZH2 (**A**) qPCR and (**B**) Western blot assays were performed to determine the expression of p15, p16, p21, p27 and p57 in BGC-823 and SGC-7901 cells after LINC00152 downregulation. (**C**) p15 and p21 mRNA levels after transfection of pcDNA or pcDNA-LINC00152 into BGC-823 and SGC-7901 cells. (**D**) p15 and p21 protein levels after transfection of pcDNA or pcDNA-LINC00152 into BGC-823 and SGC-7901 cells. (**E**) p15 and p21 mRNA levels were detected using qPCR in BGC-823 and SGC-7901 cells after si-EZH2 transfection, and protein levels (**F**) were detected using Western blots. (**G**) ChIP of EZH2 and H3K27me3 in the p15 and p21 promoter regions after siRNA treatment targeting si-NC or LINC00152-siRNA-3 in BGC-823 and SGC-7901 cells. qPCR was performed to quantify ChIP assay results. Enrichment was quantified relative to input controls. Antibody directed against IgG was used as a negative control. Results are represented as the average ± SD based on 3 independent experiments (*p < 0.05, **p < 0.01).

We then sought to determine downstream targets of LINC00152. qPCR and Western blotting confirmed that the expression of cell-cycle regulation genes p15 and p21, which are targets of EZH2 [24], was upregulated in GC cells when LINC00152 was knocked down (Figure 5A, 5B) and reduced when LINC00152 was overexpressed (Figure 5C, 5D). Upregulation of p15 and p21 was also detected when EZH2 was knocked down in BGC-823 and SGC-7901 cells (Figure 5E, 5F).

**Figure 6:**
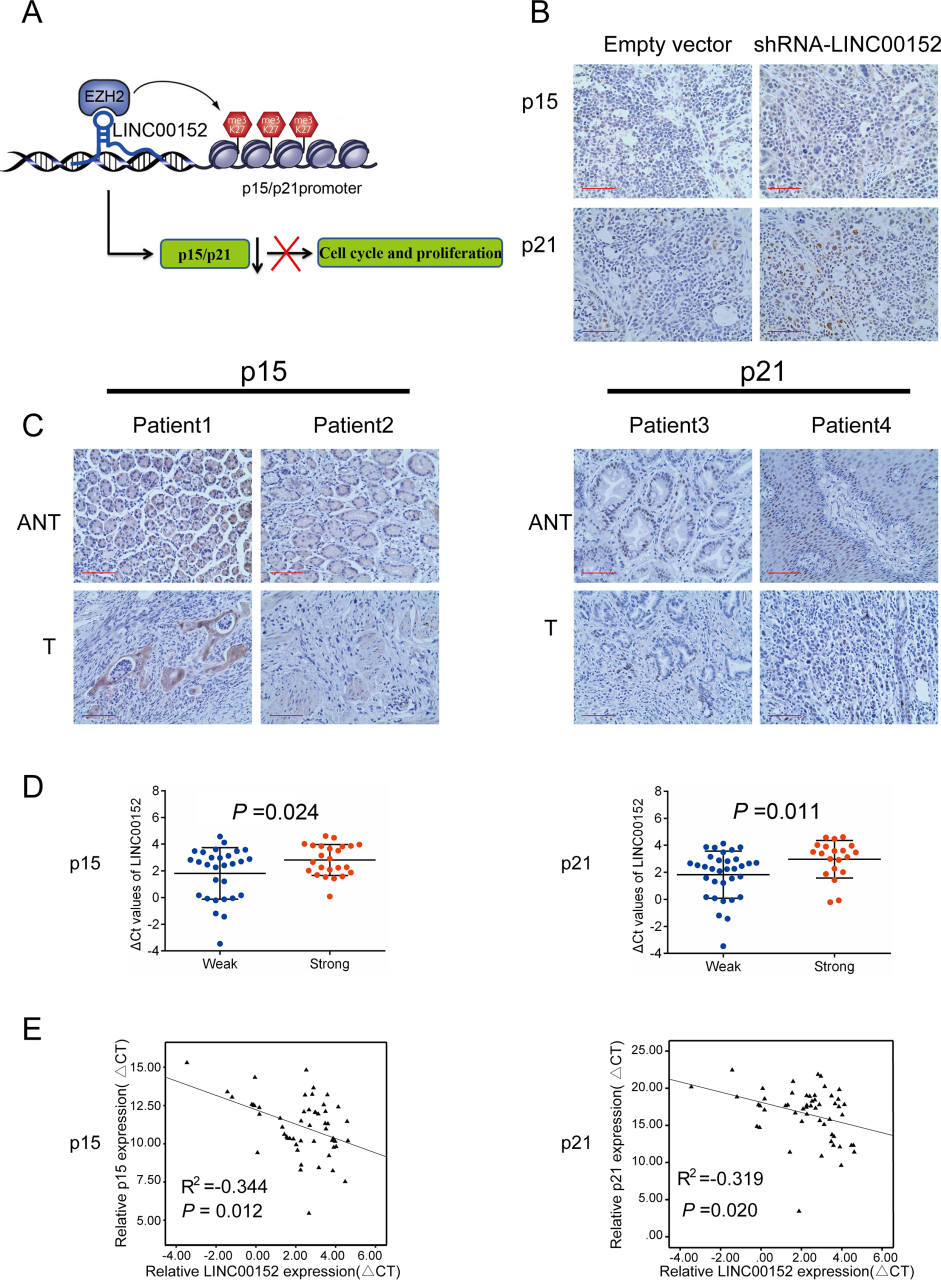
LINC00152 expression is inversely correlated with p15 and p21 levels in xenograft tumors and GC tissues (**A**) Schematic diagram of the regulation of p15 and p21 by LINC00152 and EZH2 in GC. (**B**) Representative p15 and p21 protein levels in xenograft tumors evaluated by immunohistochemistry. (**C**) p15 and p21 levels in 53 GC tissues were determined by immunohistochemistry. (**D**), (**E**) Analysis of the relationship between LINC00152 expression (ΔCT) and p15 and p21 protein (D) and mRNA levels (E) in 53 GC tissues. Error bars indicate mean ± SD. (*p < 0.05, **p < 0.01).

To determine whether LINC00152 represses transcription by recruiting EZH2 to the promoters of its target genes, we conducted Chromatin immunoprecipitation (ChIP) assays in BGC-823 and SGC-7901 cells. LINC00152 knockdown (si-RNA-3) decreased binding of EZH2 and H3K27me3 levels in both the p15 and p21 promoters compared to cells transfected with si- NC (Figure 5G). These results suggest that LINC00152 can recruit EZH2 to p15 and p21 promoters and cause epigenetic repression via trimethylation of histone H3 at lysine 27 residue (H3K27me3) (Figure 6A).

### LINC00152 is negatively correlated with p15, p21 in GC tissues

To determine the relationship between LINC00152 and p15- and p21-mediated cell-cycle regulation in GC, we measured expression of p15 and p21 using qPCR and immunohistochemistry in 53 pairs of GC and normal tissues (Figure 6C). LINC00152 expression was inversely correlated with p15 and p21 mRNA and protein levels (Figure 6D, 6E). Intriguingly, LINC00152 knockdown markedly increased levels of p15 and p21 in xenograft tumors (Figure 6B). These data suggest that LINC00152 contributes to GC development by inhibiting p15 and p21.

## DISCUSSION

Aberrant lncRNA expression plays a key role in gastric carcinogenesis [25]. LINC00152, a newly identified LinCRNA which is upregulated in GC tissues, affects cell cycle arrest, apoptosis, epithelial to mesenchymal transition (EMT), and cell migration and invasion [26]. Recently, Zhou J et al. [27] found that LINC00152 promotes tumor growth through the EGFR-mediated PI3K/AKT pathway. In addition, Ji J et al. [28] reported that LINC00152 promotes proliferation in hepatocellular carcinoma by targeting EpCAM via the mTOR signaling pathway. Together, these results indicate that LINC00152 plays a vital role in GC proliferation. However, the detailed molecular mechanisms through which LINC00152 regulates cell cycle transitions remain unknown. Our results suggest that LINC00152 may promote cell proliferation by accelerating cell cycle progression.

Regulation of the cell cycle is important in cellular proliferation, and loss of cell cycle control is involved in carcinogenesis [29]. In mammalian cells, the G1–S transition is controlled by cyclins, cyclin-dependent kinases (CDKs), and CDK inhibitors (CKIs) [30]. p15 and p21 are two important CKI family members and are frequently dysregulated in cancers [31]. Both p15 and p21 inhibit cell cycle progression and, therefore, cell growth [32]. Here, we provide the first evidence that LINC00152 promotes cell proliferation by downregulating p15 and p21 expression in GC. Our data indicate that aggressive GC cells are characterized by higher LINC00152 expression, and that increased LINC00152 levels accelerate the progression of gastric cancer.

We also sought to determine the underlying molecular mechanisms by which LINC00152 regulates downstream effectors in GC. We focused on the PRC2 subunit EZH2 because other lncRNAs regulate downstream effectors via EZH2-driven H3K27 methylation [33]. Indeed, we found that LINC00152 binds to EZH2 and recruits it to the promoter regions of its target genes in GC. Unfortunately, the association between LINC00152 and SUZ12, another crucial component of PRC2 [15], was not verified here. This may be explained by a lower binding affinity of SUZ12 for lncRNA compared to EZH2 [34]. In addition, the binding of SUZ12 to RNA requires a unique stem–loop structure in the RNA [35]. The RepA protein shares this property, and computer analysis of the SUZ12 amino acid sequence revealed a domain at its N-terminus that requires this stem-loop conformation for RNA binding [36]. It is possible that LINC00152 does not possess the stem-loop structure that would allow biding to SUZ12. Furthermore, a recent paper reported that PRC2-RNA interactions change dramatically depending on specific experimental conditions [37].

lncRNAs have four known molecular functions: signal, decoy, guide, and scaffold [38]. Our findings suggest that LINC00152 primarily plays the guide role in GC. LINC00152 recruits EZH2 to the p15 and p21 promoters, resulting in the silencing of those genes. However, individual lncRNAs can have more than one molecular function [38], and LINC00152 may play different roles in other cancer types [27, 28]. For example, the well-studied lncRNA HOTAIR acts as a scaffold by connecting PRC2 to the LSD1/CoREST/REST complex, and the HOTAIR/PRC2/LSD1 complex as a whole suppresses gene expression [39]. HOTAIR also functions as a decoy by acting as a sink for miR-331–3p in GC [40]. In addition to upregulating cell cycle progression by inhibiting p15 and p21, EZH2 can regulate neural progenitor cell proliferation by suppressing Pten expression and activating Akt-mTOR pathway [41]. Together, these reports indicate that LINC00152 might be a key regulator in the development of cancer because of its ability to increase EZH2-dependent gene silencing.

Here, we show for the first time that LINC00152 promotes tumor cell cycle progression by binding to EZH2 and repressing p15 and p21 in GC. However, LINC00152 may influence GC progression on other ways as well. For example, in addition to promoting cell cycle progression, LINC00152 might promote tumor cell invasion and metastasis by repressing E-cadherin via EZH2 in GC. This hypothesis is supported by a previous report that E-cadherin protein was upregulated following LINC00152 knockdown [26]. EZH2 could also mediate transcriptional silencing of E-cadherin though trimethylation of H3 lysine 27 [42, 43]. Future studies of LINC00152 should investigate these mechanisms directly to determine whether they contribute to GC progression.

Collectively, our results clearly identified elevated LNC00152 expression in GC tissues and a novel oncogenic role for LNC00152 in GC. The loss of LNC00152 inhibited GC cell proliferation *in vitro* and suppressed tumor growth *in vivo*, likely by recruiting EZH2 to the p15 and p21 promoters and inhibiting their expression. Our findings provide additional insight into molecular mechanisms of gastric carcinogenesis. Its role in gastric cancer progression suggests that LINC00152 may prove a useful biomarker and therapeutic target for GC treatment.

## MATERIALS AND METHODS

### Bioinformatics analysis

Gastric cancer gene expression data was obtained from the NCBI GEO, (http://www.ncbi.nlm.nih.gov/geo/). Three data sets were included: GSE13861, GSE13911, and GSE26253. GSE13861 consisted of 60 primary gastric adenocarcinoma tissues and 19 surrounding normal fresh frozen tissues. All the tissues were obtained after curative resection and pathologic confirmation at Yonsei Cancer Center (Seoul, Korea, YGC cohort). GSE13911 consisted of 38 gastric cancer tissues and 31 normal adjacent tissues obtained from a series of GC cases identified in an area around Florence (Italy, Italy cohort). GSE26253 consisted of 432 cases of stage IB–IV GC treated with standard chemoradiotherapy after curative resection at Samsung Medical Center (Seoul, South Korea, Samsung cohort), along with survival data. GSE13861 and GSE26253 were normalized data. The raw CEL files from the Affymetrix HG-U133 plus 2.0 arrays (Affymetrix, Santa Clara, CA, USA) for GSE13911 were processed and normalized using the Robust Multichip Average (RMA) as previously described [44]. After downloading probe sequences from GEO or microarray manufacturers, we use BLAST+ 2.2.30 to re-annotate probes on GENCODE Release 21. For multiple probes corresponding to one lncRNA, the maximum normalized signal was selected.

lncRNA expression profiles were generated using the significance analysis of microarrays (SAM) method in MeV software (http://www.tm4.org/mev.html). The thresholds for upregulated and downregulated genes were ≥ 2− or ≤ 0.5-fold changes, and we controlled the delta value so that the median number of false significant genes remained at zero. Two-class paired or two-class unpaired comparisons were used according to experimental design.

To study the functional roles of LINC00152 in GC pathogenesis and the association between LINC00152 and EZH2, a GSEA was performed as previously described [45]. GSEA version 2.0 from the Broad Institute at MIT was used. The GSE15459 dataset was analyzed using GSEA. GSE15459 consisted of 200 primary gastric cancers from the Singapore. Probes were re-annotated as described above. To collapse each probe set on the array to a single sequence, the probe with the highest variance that corresponded to each gene was selected, which produced a 22,937 (genes) × 200 (GCs) expression matrix. 200 GC patients were classified into two groups (low or high group) based on the median LINC00152 level ratio. Gene sets with named oncogenic signatures from the Molecular Signatures Database (MSigDB, http://www.broadinstitute.org/gsea/msigdb/index.jsp) were used as functional gene sets for GSEA. As a metric for ranking genes in GSEA, signal-to-noise ratios between the high and low LINC00152 sample expression means were used, and all other parameters were set to default values. Gene sets with an FDR of 0.25, a well-established cutoff for the identification of biologically relevant genes, were considered enriched.

### Tissue collection and ethics statement

A cohort of 97 primary GC patients who had surgery at First Affiliated Hospital of Nanjing Medical University between 2008 and 2009 (Nanjing cohort, China) were enrolled in this study. None received chemotherapy or radiotherapy prior to surgery. This study was approved by Ethics Committee of Nanjing Medical University. All patients participated after providing informed consent. Tumor stage was evaluated in accordance with the tumor-node-metastasis (TNM) classification system UICC/AJCC 2002. Clinical characteristics are shown in Table 1. Patients discharged from the hospital were routinely followed up with at least once a year according to a scheduled program.

### Cell lines and culture conditions

Human gastric cancer cell lines (MGC-803, SGC-7901, BGC-823, and AGS) and a normal gastric epithelium cell line (GES-1) were obtained from Chinese Academy of Sciences Committee on Type Culture Collection cell bank. Cells were cultured in RPMI 1640 or DMEM (GIBCO-BRL) medium supplemented with 10% FBS (Invitrogen), 100 U/mL penicillin, and 100 mg/mL streptomycin (invitrogen) in incubator at 37°C with 5% CO_2_.

### RNA extraction and qPCR analyses

Total RNA was extracted from frozen tissues using TRIzol (Invitrogen) according to the manufacturer's instructions. RNA concentrations were estimated by spectrophotometer absorbance readings at 260 nm. One μg of total RNA was reverse transcribed to cDNA using a Reverse Transcription Kit (Takara). Real-time PCR was performed with SYBR Green (Takara, Dalian China). GAPDH was used as reference for mRNA or lncRNAs. Primer sequences in our study are listed in Supplementary Table S1. Relative expression was calculated using the equation: ΔCt = Ct (target gene) – Ct (GAPDH), fold expression = 2^−(ΔCt(tumor) – ΔCt(normal))^ [46].

### Subcellular fractionation

To determine the cellular localization of LINC00152, cytoplasm and nuclear fractions were collected according to the manufacturer's instructions for the PARIS Kit (Life Technologies, Carlsbad, CA, USA).

### Cell transfection

LINC00152 siRNA, EZH2 siRNA, and negative control siRNA (si-NC) were purchased from Invitrogen (Invitrogen, USA). siRNA sequences are listed in Supplementary Table S1. Full-length complementary DNA for LINC00152, shRNA-LINC00152, and empty vector was synthesized by Realgene (Nanjing, China) and subcloned into the pcDNA3.1 (+) vector (Invitrogen) according to the manufacturer's instructions. The resulting construct was verified using sequencing. BGC-823 or SGC-7901 cells were grown in 6-well plates until confluent, then transfected with Lipofectamine 2000 (Invitrogen, Shanghai, China) according to the manufacturer's instructions. At 48 h post transfection, cells were harvested for qPCR or Western blot analysis.

### Cell proliferation analysis *in vitro*

Cell proliferation was measured using a Cell Proliferation Reagent Kit I (MTT) (Roche). 3000 transfected cells were plated in each well of 96-well plates and proliferation was quantified every 24 hours according to the manufacturer's instructions. For the colony formation assay, transfected cells were placed in each well of 6-well plates and maintained in proper media containing 10% FBS for two weeks, and medium was replaced every 3 days. Colonies were then fixed with methanol and stained with 0.1% crystal violet (Sigma) in PBS for 15 minutes. Colony formation was determined by counting the number of stained colonies. The EdU assay was performed after cells were cultured for 48 h. Finally, the cells were washed, mounted with mounting medium containing DAPI (Santa Cruz Biotechnology, CA, USA), and imaged using an Olympus FV1000 confocal microscope (Olympus, NY, USA).

### Tumor formation assay in a nude mouse model

SGC-7901 cells were transfected with sh-LINC00152 or empty vector using Lipofectamine 2000 (Invitrogen). After 48 h, cells were collected and injected into either side of the posterior flank of the male BALB/c nude mice (4–5 weeks old). Mice were purchased from Shanghai Experimental Animal Center of the Chinese Academy of Sciences. Tumor volumes and weights were measured every 4 days in mice in the control (6 mice) and sh-LINC00152 (6 mice) groups, and tumor volumes were calculated by using the equation V = 0.5 × D × d^2^ (V, volume; D, longitudinal diameter; d, latitudinal diameter). Sixteen days after injection, the mice were killed and tumor weights were measured. Primary tumors were excised and tumor tissues were used to perform qPCR analysis of LINC00152 levels and immunostaining analysis of Ki-67 protein expression. This study was carried out strictly in accordance with the recommendations in the Guide for the Care and Use of Laboratory Animals of the National Institutes of Health. The protocol was approved by the Committee on the Ethics of Animal Experiments of the Nanjing Medical University (Permit Number: 200933).

### Cell-cycle analysis

Transfected cells were harvested 48 h after transfection. The cells were fixed in 70% ethanol, washed once with PBS, and then labeled with propidium iodide (Sigma-Aldrich) in the presence of RNase A (Sigma-Aldrich) for 30 min in the dark (50 g/mL). Samples were run on a FACScan flow cytometer (Becton-Dickinson, FL, NJ, USA), and the percentages of cells within each phase of the cell cycle were analyzed using Cell Quest software.

### Western blot assay and antibodies

Cells protein lysates were separated by 15% SDS-polyacrylamide gel electrophoresis (SDS-PAGE), transferred to 0.22 μm NC membranes (Sigma), and incubated with specific antibodies. GAPDH antibody was used as control.

Autoradiograms were quantified by densitometry (Quantity One software; Bio-Rad). Anti-p15 was purchased from Santa Cruz Biotechnology, Inc, and anti-p21 was purchased from Cell Signaling Technology.

### RIP

RIP experiments were performed using a Magna RIP™ RNA-Binding Protein Immunoprecipitation Kit (Millipore, USA) according to the manufacturer's instructions. Antibody for EZH2 RIP assays was from Abcam.

### RNA pull-down

RNA pull-down was performed using a Magnetic RNA-Protein Pull-Down Kit (Pierce Biotechnology, USA) in accordance with the manufacturer's instructions.

### ChIP assays

ChIP assays were performed using an EZ-CHIP KIT according to the manufacturer's instructions (Millipore, USA). EZH2 antibody was obtained from Abcam. H3 trimethyl Lys 27 antibody was from Millipore. The ChIP primer sequences are listed in Supplementary Table S1. Quantification of immunoprecipitated DNA was performed using qPCR with SYBR Green Mix (Takara). ChIP data is presented as a percentage relative to the input DNA using the equation 2^[Input Ct- Target Ct]^ ×0.1 × 100 [19].

### Immunohistochemistry (IHC)

Immunohistochemical analysis was conducted as previously described [47].

### Statistical analysis

All statistical analyses were performed using SPSS 21.0 software (IBM, SPSS, Chicago, IL, USA). The statistical significance of differences between groups was determined using a Student's *t*-test, χ2 test, Fisher's exact test, Mann-Whitney test, Kruskal-wallis test, or Wilcoxon test, as appropriate. Survival curves were estimated using the Kaplan–Meier method. The log-rank test was used to identify statistically significant differences between survival curves. Cox proportional hazards analysis was performed to calculate the hazard ratio (HR) and the 95% confidence interval (CI) when evaluating the association between LINC00152 expression and overall survival time. A multivariate Cox regression was performed to adjust for other covariates. Spearman correlation analyses were performed to investigate correlations between LINC00152 and p15 and p21 mRNA expression. Categorical data was analyzed using chi-squared tests. A receiver operating characteristic (ROC) curve was produced to determine the diagnostic value of LINC00152 in GC and adjacent non tumor tissues. A two-tailed *p* value of 0.05 or less was considered statistically significant. All graphs were plotted using GraphPad Prism 6.0 (GraphPad Software, La Jolla, CA).

## SUPPLEMENTARY MATERIALS FIGURES AND TABLE


