# Supplementary material for: Long intergenic non-coding RNA 00152 promotes tumor cell cycle progression by binding to EZH2 and repressing p15 and p21 in gastric cancer
Source: Oncotarget. 2016 Jan 19;7(9):9773–87. doi: 10.18632/oncotarget.6949 (PMC4891083; doi:10.18632/oncotarget.6949)
Supplement: Supplementary file 1 [file oncotarget-07-09773-s001.pdf]

Long intergenic non-coding RNA 00152 promotes tumor cell cycle progression by binding to EZH2 and repressing p15 and p21 in gastric cancer

Supplementary Materials

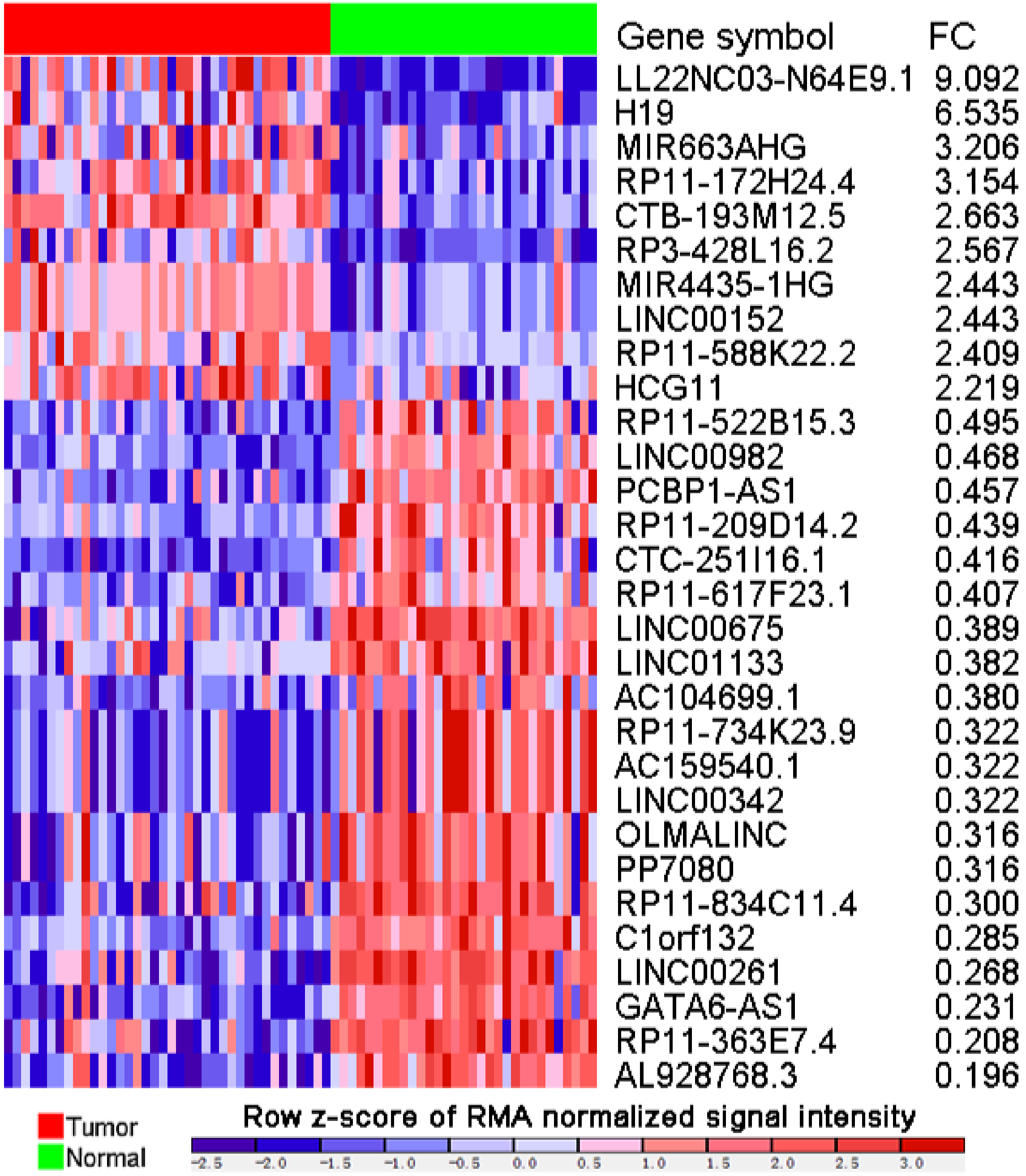

Supplementary Figure S1: Clustering heatmap of GSE13911 based on 30 differentially expressed long non-coding RNAs. Each column represents one sample and each row represents one long non-coding RNA. Red indicates high, and blue indicates low, gene expression.

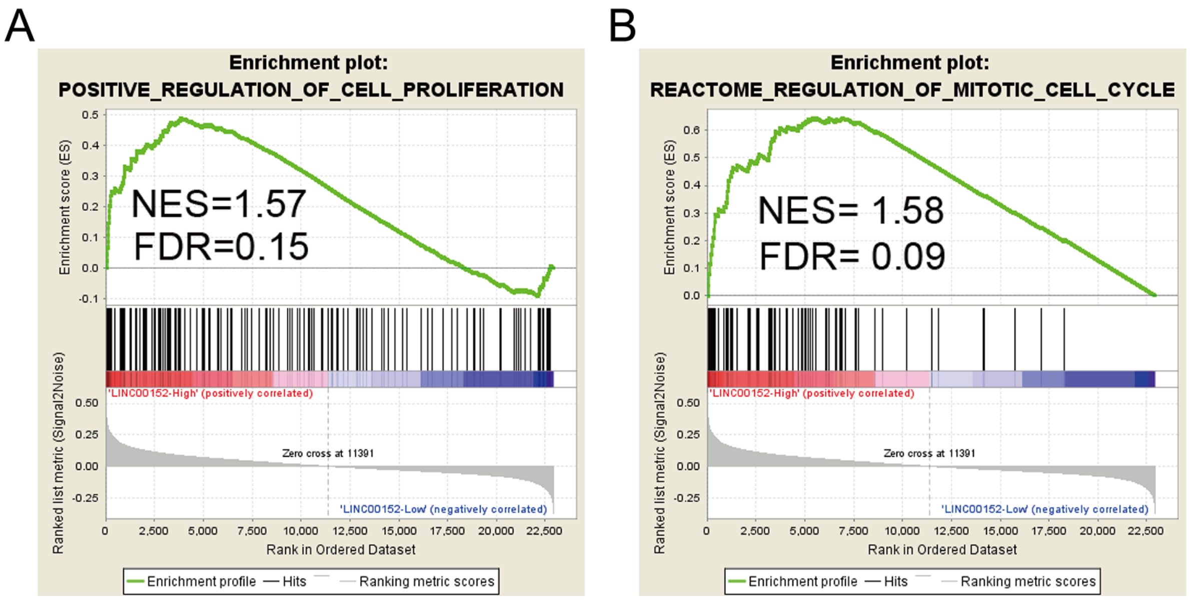

Supplementary Figure S2: GSEA in high and low LINC00152 expression groups. GSEA comparing the high (red) and low (blue) LINC00152 expression GC patient groups in the GSE15459 dataset, illustrating biologic processes in both subgroups. Enrichment maps were used for visualization of the GSEA results. Enrichment plots are shown for a set of activated genes related to cell proliferation (**A**) and cell cycle regulation (**B**) in the GSE15459 dataset. The enrichment score (ES, green line) indicates the degree to which the gene set is overrepresented at the top or bottom of the ranked list of genes. Black bars indicate the position of genes in the ranked list of genes included in the analysis. A positive value indicates stronger correlation with high LINC00152 expression and a negative value indicates stronger correlation with low LINC00152 expression.

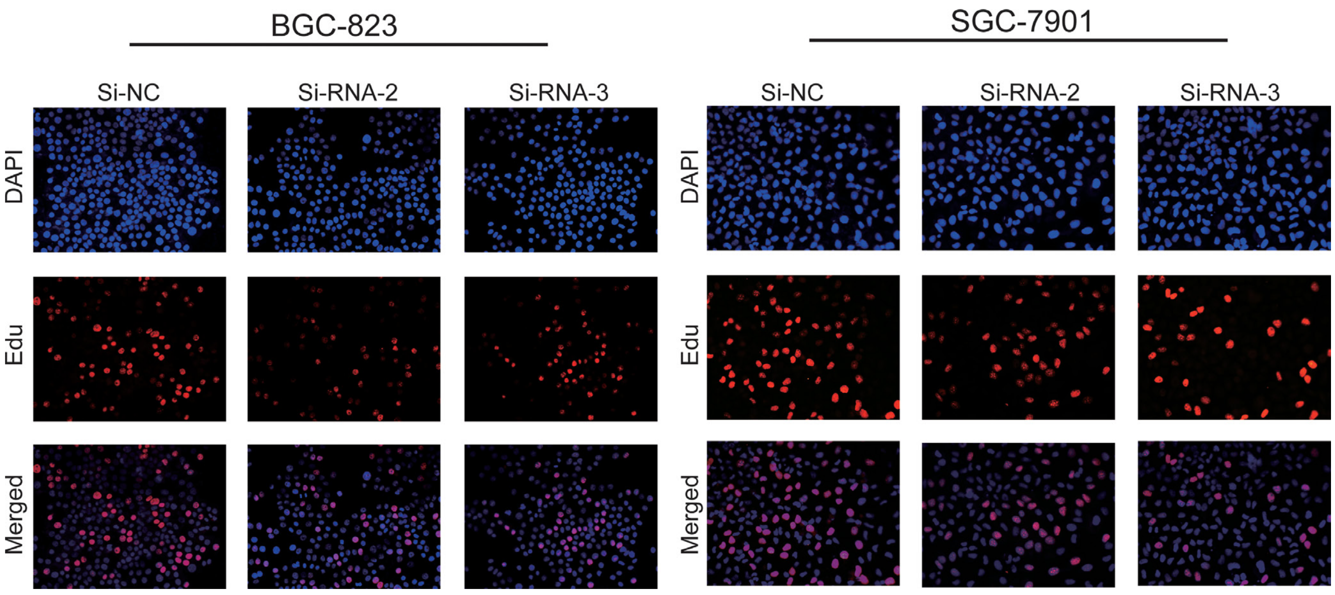

Supplementary Figure S3: Proliferation in BGC-823 and SGC-7901 cells treated with LINC00152 siRNA or negative control. EdU (red)/DAPI (blue) immunostaining confirmed that cell proliferation decreased following LINC00152 knockdown.

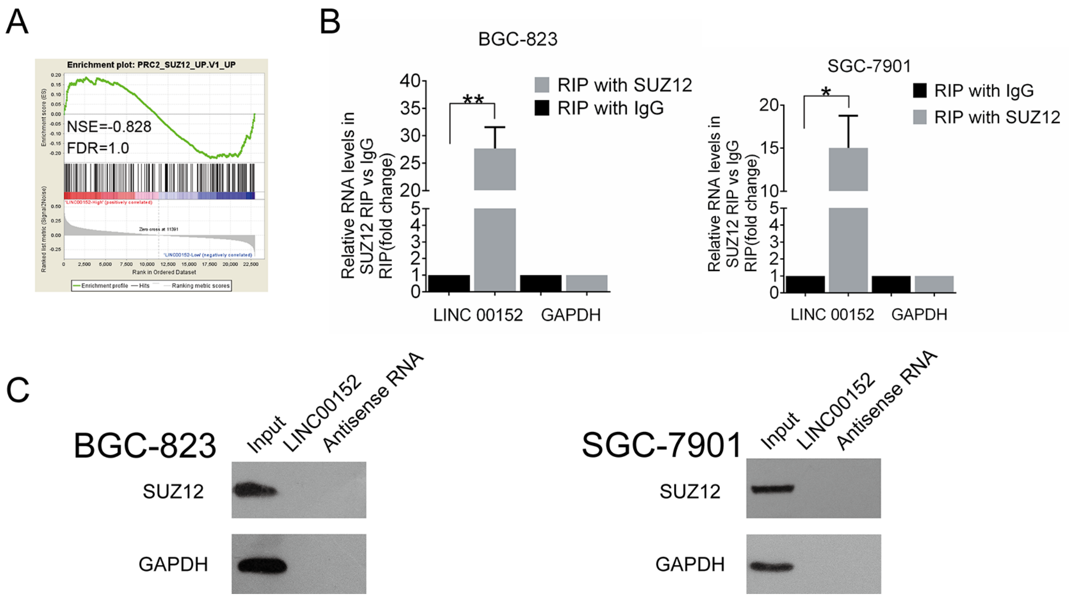

Supplementary Figure S4: Analyses of the association between LINC00152 and SUZ12. (**A**) GSEA analyses of the correlation between LINC00152 and SUZ12 expression. (**B**) An RIP assay was performed using an SUZ12 antibody. (**C**) RNA pull-down experiment in BGC-823 and SGC-7901 cells.

Supplementary Table S1: The list of primers and the sequence of siRNAs

GENE	Forward primer	Reverse primer
**human qPCR**		
**LINC00152**	5′AAAATCACGACTCAGCCCCC 3′	5′AATGGGAAACCGACCAGACC 3′
**P15**	5′GGACTAGTGGAGAAGGTGCG 3′	5′GGGC GCTGCCCATCATCATG 3′
**P16**	5′CACCGAATAGTTACGGTCGG 3′	5′GCACGGGTCGGGTGAGAGTG 3′
**P21**	5′AGACCATGTGGACCTGTCACTG3′	5′GTTTGGAGTGGTAGAAATCTGTC3′
**P57**	5′AGCTGCACTCGGGGATTT3′	5′GGGCTCTTTGGGCTCTAA 3′
**EZH2**	5′TGCACATCCTGACTTCTGTG 3′	5′AAGGGCATTCACCAACTCC 3′
**U6**	5′CTCGCTTCGGCAGCACA 3′	5′AACGCTTCACGAATTTGCGT3′
**GAPDH**	5′GGGAGCCAAAAGGGTCAT3′	5′GAGTCCTTCCACGATACCAA 3′
**siRNA**		
**si-LINC00152RNA-1**	5′UGAUCGAAUAUGACAGACACCGAAA3′	5′UUUCGGUGUCUGUCAUAUUCGAUCA3′
**si-LINC00152RNA-2**	5′CAGGGAAUCUUUCAGCUGGAUUCCG3′	5′CGGAAUGCAGCUGAAAGAUUCCCUG3′
**si-LINC00152RNA-3**	5′UCUAUGUGUCUUAAUCCCUUGUCCU3′	5′AGGACAAGGGAUUAAGACACAUAGA3′
**si-EZH2**	5′GAGGUUCAGACGAGCUGAUUU3′	5′AUCAGCUCGUCUGAACCUCUU3′
**sh-LINC00152**	5′CACCGCACACTTGATCGAATAT GACATTCAAGAGATGTCATATTC GATCAAGTGTGTTTTTTG3′	5′GATCCAAAAAACACACTTGATCGA ATATGACATCTCTTGAATGTCATATTC GATCAAGTGTGC3′
**ChIP qPCR primers**		
**P15**	5′TCTGGTAAGGGTGTGCTGTG3′	5′AAAACTCCTCTGTGGCATGTG3′
**P21**	5′GGTGTCTAGGTGCTCCAGGT3′	5′GCACTCTCCAGGAGGACACA3′
